# Lipoproteins and Their Effects on the Cardiovascular System

**DOI:** 10.7759/cureus.48865

**Published:** 2023-11-15

**Authors:** Pratyush Das, Nishikant Ingole

**Affiliations:** 1 Medicine, Jawaharlal Nehru Medical College, Datta Meghe Institute of Higher Education and Research, Wardha, IND; 2 Pharmacology, Jawaharlal Nehru Medical College, Datta Meghe Institute of Higher Education and Research, Wardha, IND

**Keywords:** myocardial infarction, atherosclerosis, lipid metabolism, cardio vascular disease, high density lipo-protein cholesterol

## Abstract

Coronary heart disease is the foremost leading cause of death across the world. It mainly involves the blood vessels, which supply the heart. Plaque formation due to lipid deposition leads to the narrowing of the vessels, obstructing blood flow. Therefore, lipoproteins such as high-density lipoproteins (HDL), low-density lipoproteins (LDL), very low-density lipoproteins (VLDL), and chylomicrons play a crucial role in cardiovascular diseases. Lipoproteins are carrier molecules made up of proteins and fats. They carry cholesterol through the bloodstream and transport it to the peripheral tissues or the liver. There are several classes of lipoproteins in the blood, namely HDL, LDL, VLDL, and chylomicrons. Depending on the lipoproteins, an excess of them can either harm or benefit the body. Low-density lipoprotein, nicknamed 'the bad cholesterol,' transports fatty molecules from the liver and deposits them in peripheral tissues or central vessels. Thus, excess LDL can cause blockage of the arteries supplying major organs. High-density lipoprotein, nicknamed 'the good cholesterol,' transports the excess fatty molecules to the liver for their metabolism and removal from the body. Hence, high levels of HDL are an indication of a healthy body. Thus, lipoproteins are important molecules, and their proper regulation is essential to maintaining a healthy body. An effective way to maintain a balanced lipoprotein level is to have a properly balanced diet with high protein and low fat. Regular exercise, both indoors and outdoors, is recommended. If cholesterol levels are not maintained by diet and exercise, medication is advised after consulting medical experts. This review aims to inform people about lipoproteins, their importance, and maintaining a healthy lipoprotein level.

## Introduction and background

Lipoproteins are essential molecules that transport lipids, such as cholesterol and triglycerides, throughout the body. These are complex and dynamic structures that act as carriers, facilitating the movement of lipids through the bloodstream to various tissues and organs according to their needs. The interaction of lipoproteins in our physiology is critical because it directly affects our general health and well-being. These remarkable molecules have an ingenious design and contain a hydrophobic lipid core protected by a hydrophilic outer layer of proteins and phospholipids. This ingenious arrangement allows lipoproteins to move through the aqueous environment of our bloodstream without clumping or precipitation. Lipoproteins are classified according to their density, which is determined by the proportion of lipids in the proteins they carry [1].

Lipoproteins are categorized into four primary classes: low-density lipoproteins (LDL), chylomicrons, very low-density lipoproteins (VLDL), and high-density lipoproteins (HDL). Each of these classes performs different functions and plays essential roles in lipid metabolism. Lipoproteins are crucial for various bodily functions and also contribute to the development of specific health conditions. Elevated levels of LDL cholesterol, often referred to as 'bad cholesterol,' are linked to an elevated risk of atherosclerosis and cardiovascular diseases. Conversely, higher HDL cholesterol levels, known as 'good cholesterol,' offer protection against cardiovascular problems [2].

The understanding of the workings of lipoproteins has significant implications for medical research and clinical practice. Researchers continuously explore new avenues to manage lipid disorders, develop treatments for cardiovascular diseases, and optimize lipid transport to enhance overall health. This exploration of lipoproteins delves into their structure, functions, significance in lipid metabolism, and implications for human health and disease. By gaining a deeper understanding of lipoproteins, we can appreciate the remarkable complexity of our biological systems and work towards promoting better health outcomes for individuals worldwide [3].

## Review

Methodology

In July 2023, we conducted a comprehensive search through PubMed and Google Scholar, using specific keywords such as "lipoproteins" and "cardiovascular diseases." The search criteria included terms like "low-density lipoprotein" and "very low-density lipoprotein" (in title or abstract) or their abbreviated forms "LDL" and "VLDL." Additionally, we employed medical subject headings (MeSH) terms like "atherosclerosis" to refine our search. We also incorporated terms related to different age groups, such as "young adults" and "geriatric population," as well as "Quality of Life (QoL)." To ensure a comprehensive search, we cross-referenced critical references from the bibliographies of relevant studies. This search was updated in August 2023.

**Figure 1:**
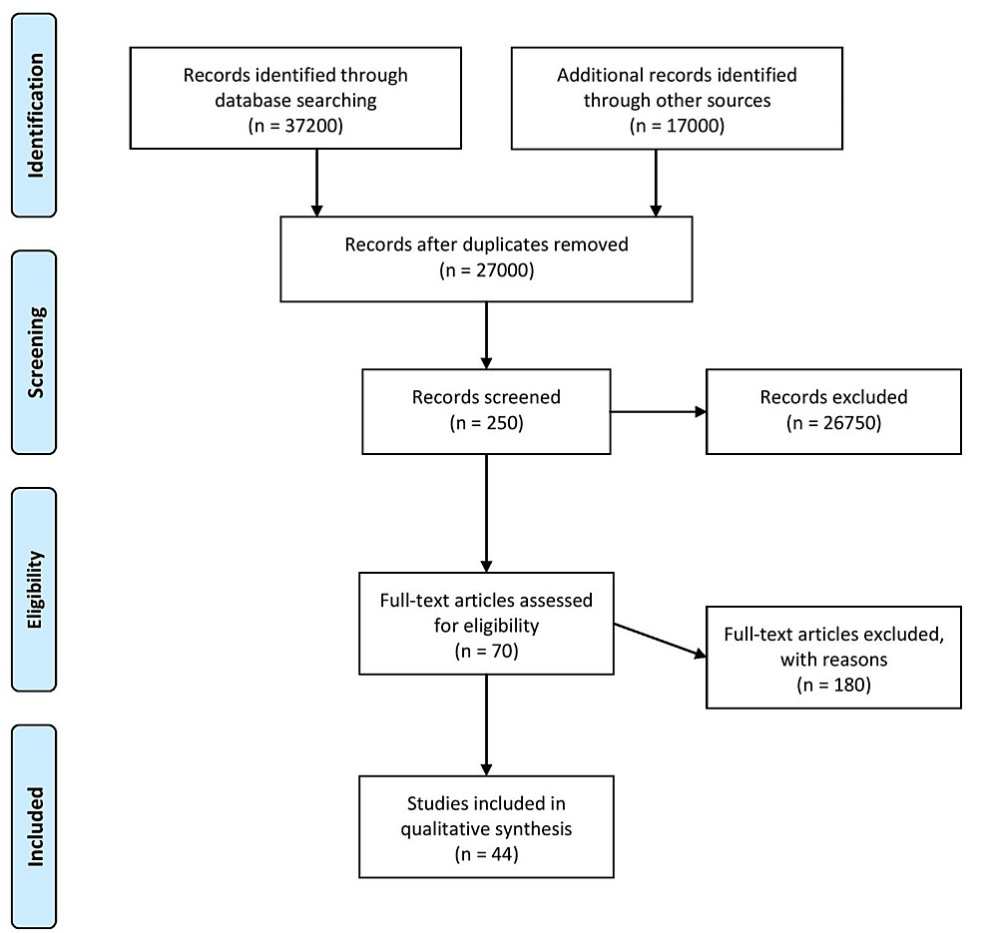
PRISMA flow diagram PRISMA: Preferred Reporting Items for Systematic Reviews and Meta-Analyses

In total, we examined 37,200 articles to investigate the impact of lipoproteins on the cardiovascular system. Our search strategies involved a combination of the terms "lipoproteins" and "cardiovascular diseases." We applied filters to include only clinical trials, meta-analyses, randomized control trials, and systematic reviews. To ensure the quality of our selection process, one reviewer (PD) initially screened the retrieved studies based on the title and abstract, while the other reviewer (NI) independently reviewed approximately 20% of these studies to validate their inclusion. Any discrepancies in the selection process were resolved through discussion. Figure 1 shows the methodology in the form of a Preferred Reporting Items for Systematic Reviews and Meta-Analyses (PRISMA) flow diagram.

Review

Lipoproteins are intricate macromolecular complexes characterized by a central hydrophobic core, predominantly rich in triglycerides and cholesterol esters. The central core is surrounded by a hydrophilic membrane composed of apolipoproteins, free cholesterol, and phospholipids. Within the circulatory system, these plasma lipoproteins can be separated into five principal categories based on their dimensions, lipid constituency, and apolipoprotein content, i.e., HDLs, LDLs, intermediate-density lipoproteins (IDLs), VLDLs, and chylomicrons [4]. Table 1 shows different classes of lipoproteins.

**Table 1:** Classes of lipoprotein and its characteristics

Lipoproteins	Density (g/ml)	Size (nm)	Major Lipids
Chylomicrons	<0.930	75-1200	Triglycerides
Chylomicrons remnants	0.930-1.006	30-80	Triglycerides, Cholesterol
Very low-density lipoproteins	0.930-1.006	30-80	Triglycerides
Low-density lipoproteins	1.019-1.063	18-25	Cholesterol
Intermediate-density lipoproteins	1.006-1.019	25-35	Triglycerides, Cholesterol
High-density lipoproteins	1.063-1.210	5-12	Cholesterol, Phospholipids

Chylomicrons

Chylomicrons are large and spherical lipoprotein particles that are formed in intestinal cells after the absorption and digestion of dietary fats. These lipoprotein particles are essential in transporting lipid particles from the small intestine to tissues throughout the body. Chylomicron particles comprise a central core composed mainly of triglycerides derived from degraded fats, surrounded by a thin single layer containing phospholipids, free cholesterol, and apolipoproteins [5]. After the formation of chylomicrons in the intestinal cells, they enter the lymph nodes and the bloodstream via the thoracic duct. Due to their large size and high triglyceride content, chylomicrons give the blood a milky appearance after a fatty meal, commonly known as chyle. Chylomicrons that enter the bloodstream move to various tissues and interact with the enzyme lipoprotein lipase on the surface of blood vessels [6]. The primary function of lipoprotein lipase is to break down triglycerides present within chylomicrons into free fatty acids and glycerol, which are then absorbed by various tissues for energy production or storage. After removing most triglycerides, chylomicrons become more minor remnants, eventually cleared by the liver. This process is essential for delivering dietary lipids to tissues for energy and other metabolic needs. Any disruption in chylomicron metabolism can lead to disorders like familial chylomicronaemia syndrome, characterized by extremely high levels of triglycerides in the blood, posing a threat of pancreatitis [7].

High-density lipoproteins

High-density lipoproteins are a class of lipoproteins found in the bloodstream and play a crucial role in lipid metabolism. They are often called 'good cholesterol' due to their protective effects on cardiovascular health [8]. These particles are smaller and denser than other lipoproteins and consist of a hydrophilic shell surrounding a hydrophobic core, like other lipoprotein classes. High-density lipoprotein particles are primarily formed in the liver and intestine but can also be generated in peripheral tissues like the small intestine, adipose tissue, and macrophages. Apolipoproteins, especially ApoA-I, are the principal structural proteins of high-density lipoproteins and play an essential role in stabilizing the particle and mediating its interactions with enzymes and cell receptors. High-density lipoproteins transport excess cholesterol back to the liver from peripheral tissues, including arterial walls, to be excreted in bile [9]. This process helps prevent cholesterol from building up in blood vessels, reducing the risk of all significant cardiovascular diseases. High-density lipoproteins also have anti-inflammatory, antioxidant, and antithrombotic effects, further contributing to their cardioprotective properties [10]. It helps maintain endothelial function and prevents LDL oxidation, promoting atherosclerotic plaque formation. In addition, HDLs can increase nitric oxide production, a vasodilator that improves blood vessel flexibility and lowers blood pressure. Higher levels of HDL cholesterol in the blood are associated with a reduced prospect of cardiovascular disease.

In contrast, a low level of HDL (hypoalphalipoproteinemia) is considered a risk factor for heart disease. Lifestyle habits such as regular exercise, a balanced diet, and moderate alcohol consumption can raise the level of HDLs. However, genetic factors can also affect HDL metabolism and its levels [11].

Low-density lipoproteins

Low-density lipoproteins are a class of circulating lipoproteins that carry cholesterol and triglycerides from the liver to peripheral tissues throughout the body. Low-density lipoproteins are often referred to as 'bad cholesterol' because elevated levels in the blood are associated with an increased risk of cardiovascular disease [12]. Low-density lipoprotein particles are more visible and less dense than lipoproteins, such as HDLs. They have a central hydrophobic core composed of cholesterol esters and triglycerides. A single-layer membrane of free cholesterol, apolipoproteins, and phospholipids surrounds the core. The primary apolipoprotein forming LDLs is ApoB-100 [13]. The principal function of LDLs is to move cholesterol to cells that need it for various purposes, including the synthesis of cell membranes, hormones, and bile acids.

However, excessive LDLs can cause cholesterol to build up in artery walls, triggering atherosclerosis, a condition characterized by plaque buildup in blood vessels. This formation or buildup of plaque can cause narrowing and hardening of the arteries, leading to reduced blood flow and an increased risk of myocardial infarction and strokes [14]. Low-density lipoprotein cholesterol can undergo oxidative changes, damaging blood vessels more. Macrophages in the arterial wall take up oxidized LDL particles, forming foam cells, a critical step in atherosclerotic plaque formation.

Many factors contribute to high LDL cholesterol, including genetics, diet, physical activity, and lifestyle choices. Diets high in saturated and trans fats and excessive caloric intake can cause a chronic rise in LDL levels. On the other hand, a balanced diet rich in green vegetables, fresh fruits, and healthy fats like vegetable oil, egg whites, and red meat can help lower LDL levels and reduce cardiovascular risk [15].

Intermediate-density lipoproteins

Intermediate-density lipoproteins are a class of lipoproteins formed when metabolizing VLDLs. Intermediate-density lipoprotein particles are between VLDLs and LDLs in size and density [16]. They play a transient role in the transport of lipids in the bloodstream. Intermediate-density lipoproteins occur when VLDL particles, primarily triglycerides, interact with lipoprotein lipase. Intermediate-density lipoproteins contain a mixture of triglycerides, cholesterol esters, phospholipids, and apolipoproteins. The fate of IDLs in circulation is twofold. Some IDL particles are taken up by receptor-mediated endocytosis in the liver, where they undergo further metabolism [17]. In the liver, IDLs can be processed into LDLs by lipoprotein lipase or converted back to VLDLs for further lipid transport.

Therefore, IDLs act as a precursor to LDLs, which are often referred to as 'bad cholesterol' due to their association with the development of atherosclerosis and diseases related to atherosclerosis. An increased blood content of IDLs is usually seen in people with dyslipidemia or certain metabolic disorders. Appropriate lipid management, including dietary changes and medications, can help regulate IDL levels and maintain cardiovascular health [18].

Very low-density lipoproteins

Very low-density lipoproteins are lipoproteins produced mainly in the liver and responsible for transporting triglycerides from the liver to various tissues. Very low-density lipoprotein particles have a larger size and lower density than lipoproteins, such as LDLs and HDLs [19]. Very low-density lipoproteins are synthesized in the liver and consist of triglycerides, cholesterol, phospholipids, and apolipoproteins. Once formed, VLDLs are released into the bloodstream, serving as the primary carrier of triglycerides in adipose tissue and muscle cells for energy storage or use. In the bloodstream, VLDLs interact with the enzyme lipoprotein lipase on the surface of blood vessels [20]. Lipoprotein lipase breaks down triglycerides within VLDLs into free fatty acids and glycerol, which tissues take up for energy production or storage. As a result, the particles change their composition and become IDLs and, eventually, LDLs. Elevated levels of VLDLs in the blood are also associated with dyslipidemia, increasing the risk of atherosclerosis and cardiovascular diseases [21]. Managing VLDL levels through lifestyle changes, such as eating a balanced diet and engaging in regular physical activity, is essential for maintaining cardiovascular health.

How lipoproteins affect the Cardiovascular system

Low-Density Lipoprotein

Often referred to as 'bad cholesterol,' LDLs can significantly affect the cardiovascular system when the levels are elevated. Low-density lipoproteins play a crucial role in causing atherosclerosis. Atherosclerosis is a cardiovascular disease that occurs due to fatty-laden deposits called plaques, which build up in the inner walls of arteries [22]. Low-density lipoproteins affect the cardiovascular system in the various ways mentioned below.

Atherosclerosis: High levels of LDLs and cholesterol in the blood can lead to the accumulation of LDL deposits in the arterial walls. These LDL particles can oxidize, trigger an inflammatory response, and attract immune cells, especially macrophages, to the area. The gathering of oxidized LDLs and immune cells leads to fatty streaks, the initial phases of atherosclerotic plaques. Plaques can grow and harden with time, narrowing arteries and reducing blood flow to vital organs such as the heart, brain, and kidneys [23].

Plaque rupture: When plaques triggering atherosclerosis reach progressive stages, they become unstable and susceptible to rupture. The materials comprising it, such as cholesterol and various fatty substances, are released into the bloodstream after rupturing an already precarious plaque. These released materials instigate clot formation, further constricting already narrowed arteries. The outcome of this process may lead to either a heart attack or stroke, contingent upon the location of the plaque's development [24].

Coronary artery disease: Elevated LDL cholesterol levels play a vital role in developing coronary artery disease. This occurs when LDL deposits in the coronary arteries form plaques and decrease blood supply to the heart muscle. Ischemia to the heart causes hypoxia, leading to chest pain (angina) and a heart attack (myocardial infarction).

Peripheral arterial disease: High LDL levels can also lead to LDL deposition and plaque formation in the arteries that supply blood to the extremities, such as the legs. This condition can cause pain cramps and inhibit wound healing in the extremities [25].

**Figure 2:**
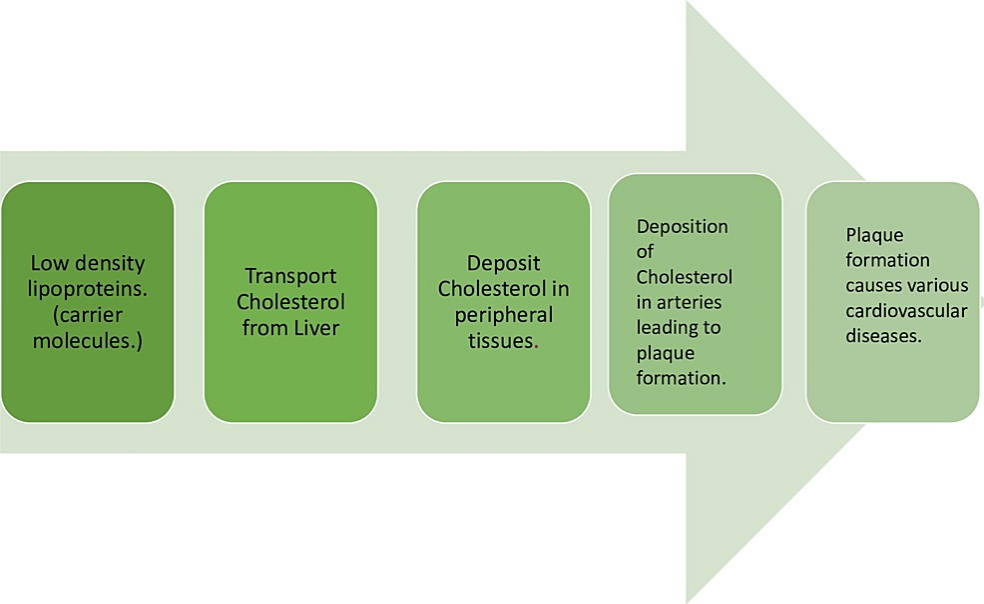
Fate of LDLs in the body and its effect on the cardiovascular system LDL: Low-density lipoprotein Image created by the author.

Hypertension: One of the main complications of LDLs other than atherosclerosis is hypertension. Higher levels of LDL cholesterol are related to an increased risk of hypertension [26]. Figure 2 depicts the impact of LDLs on the body.

High-Density Lipoprotein 

Often called 'good cholesterol,' HDL has several beneficial effects on the cardiovascular system. High-density lipoproteins are crucial for promoting cardiovascular health and protecting against heart disease. Mentioned below are some ways in which HDLs benefit the cardiovascular system.

Reverse cholesterol transport: It removes the excess cholesterol and helps prevent atherosclerosis of the arterial walls, reducing the risk of cardiovascular disease [27].

Antioxidant and anti-inflammatory properties: High-density lipoproteins have very beneficial antioxidant properties. These assist in neutralizing the destructive free radicals that oxidize LDL cholesterol [28]. These oxidized LDLs are more likely to promote atherosclerosis. In addition, HDLs have an anti-inflammatory effect that helps reduce inflammation of blood vessels. Both of these are vital processes in the prevention of atherosclerosis [29].

Vasodilation and endothelial function: High-density lipoproteins stimulate the production of nitric oxide, a potent vasodilator. Nitric oxide causes vasodilatation, which improves circulation and helps maintain normal blood pressure. High-density lipoproteins help protect endothelial cells from damage and dysfunction.

**Figure 3:**
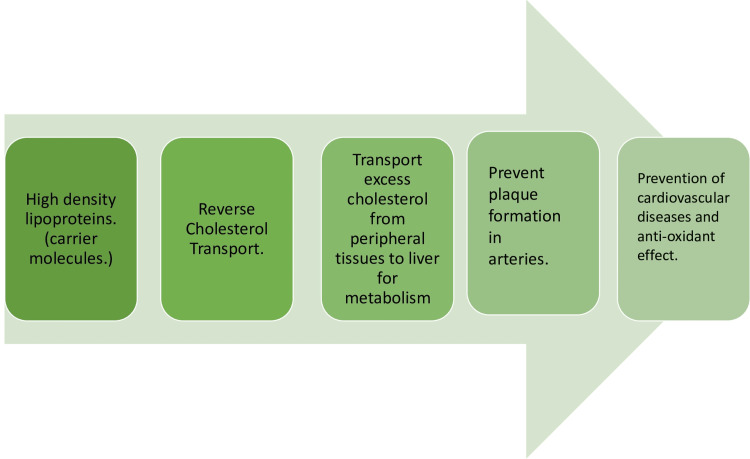
Fate of HDLs in the body and its effect on the cardiovascular system HDL: High-density lipoprotein Image created by the author

Antithrombotic effect: High-density lipoproteins help prevent thrombosis by regulating the activity of various factors involved in the clotting process. However, the extent to which it affects thrombosis is unknown [30]. Figure 3 depicts the impact of HDLs on the body.

Very Low-Density Lipoproteins

Very low-density lipoprotein significantly affects the cardiovascular system, especially at high levels. These are the primary carriers of triglycerides in circulation and play a role in lipid metabolism. Very low-density lipoproteins affect the cardiovascular system in the ways mentioned below.

Atherosclerosis: Increased levels of VLDLs can cause elevated levels of triglycerides in the blood [31]. Excess triglycerides can accumulate in the artery's walls, promoting atherosclerosis development. Very low-density lipoprotein particles transport triglycerides to peripheral tissues and leave behind residues that can cause further change, contributing to the formation of plaques.

Formation of LDLs: Very low-density lipoprotein particles are converted to IDLs when they deliver triglycerides to tissues [32]. Later, IDL particles can be further broken down to form LDL cholesterol, commonly known as 'bad cholesterol.' Increased LDL levels can cause cholesterol to build up in artery walls and promote atherosclerosis [33].

Cardiovascular disease: Dyslipidaemia characterized by high VLDL levels, is a major causative factor for cardiovascular disease. Increased VLDLs are associated with the risk of developing hypertension, atherosclerosis, coronary artery disease, myocardial infarction, and stroke.

Triglyceride-related complications: High levels of VLDLs are often associated with high triglycerides, increasing the risk of acute pancreatitis, a potentially serious condition affecting the pancreas [34].

Chylomicrons

Chylomicrons play an essential role in lipid transport and metabolism, particularly in the transport of dietary fats from the small intestine to various tissues throughout the body. However, chylomicrons are not directly implicated in cardiovascular diseases like atherosclerosis. Instead, their impact on the cardiovascular system is related to their triglycerides [35]. After a meal, chylomicrons are synthesized in the intestinal cells and enter the lymphatic system. Eventually, they are released into the bloodstream, transporting triglycerides to fatty tissue, which stores energy, and muscle tissue, which utilizes energy. Lipoprotein lipase is situated on the surface of blood vessels, facilitating the breakdown of triglycerides in chylomicrons. This enzymatic action results in the liberation of free fatty acids and glycerol, which are then available for absorption by various body tissues [36]. High levels of chylomicrons and elevated triglycerides in the blood (hypertriglyceridemia) can have several implications for cardiovascular health.

Atherosclerosis risk: Elevated triglyceride levels, especially in combination with other lipid abnormalities, are accompanied by an augmented risk of atherosclerosis. The leftovers of chylomicrons and other triglyceride-rich lipoproteins can lead to the development of atherosclerotic plaques on the endothelium of arterial walls [37].

Cardiovascular diseases: Hypertriglyceridemia is considered an independent risk factor for cardiovascular diseases, including coronary heart disease and an increased risk of heart attacks and strokes.

Pancreatitis: Extremely high levels of chylomicrons and triglycerides in the blood can lead to hypertriglyceridemia pancreatitis, characterized by pancreas inflammation, which can be life-threatening [38].

Prevention of cardiovascular diseases related to lipoproteins

Preventing lipoprotein-related cardiovascular diseases includes a blend of lifestyle modifications and medical interventions when necessary. There are critical strategies to prevent lipoprotein-related cardiovascular disease. Eat a heart-healthy diet. A dietary regimen abundant in fiber and protein, comprising elements like fruits, vegetables, whole grains, lean protein sources, and beneficial fats, can effectively manage cholesterol levels and diminish the likelihood of developing cardiovascular disease. [39]. Limiting the intake of saturated fat, trans fat, and dietary cholesterol is critical to controlling LDL cholesterol. In addition, increasing foods containing omega-3 fatty acids (e.g., fatty fish and flaxseeds) can increase HDL cholesterol and improve the overall lipid profile.

Participate in consistent physical activity. Regular exercise helps manage lipid profiles, increase HDL cholesterol, and lower triglycerides. At least 2.5 hours of moderate-intensity aerobic exercise or one hour of vigorous workout plus weight training per week is essential. And sustain a healthy weight. Obesity, especially abdominal fat, is associated with adverse changes in lipid profiles [40]. Weight loss through a healthy diet and regular exercise can significantly improve lipid levels and reduce cardiovascular risk.

Quit smoking and curb alcohol intake. Smoking damages blood vessels and promotes atherosclerosis, while excessive alcohol consumption can raise triglyceride levels. Quitting smoking and consuming moderate amounts of alcohol can be beneficial for heart health and blood vessels.

Manage diabetes and hypertension. Blood glucose levels in treating diabetes and hypertension are essential to avoid complications affecting lipoprotein metabolism. Healthcare providers can monitor lipid profiles and cardiovascular health. Early detection of lipid abnormalities allows for timely measures to prevent disease progression [41].

Medicate. Sometimes, lifestyle changes may not be enough to control lipid levels effectively. Cholesterol-lowering medications such as statins, fibrates, niacin, or bile acid sequestrants may be prescribed to lower LDL cholesterol or triglycerides, depending on individual needs.

Genetic testing and family history: Some people may have a genetic predisposition to lipids. Static knowledge of genetic history and testing can help recognize people at higher risk and allow tailored prevention and management approaches.

By taking these preventive measures, people can effectively manage lipoprotein-related risk factors and reduce the probability of cardiovascular disease, ultimately promoting heart health and overall well-being. Regular contact with health professionals is essential for personal guidance and progress monitoring [42].

Statin Intolerance and Its Management

Statins are revolutionary lipid-lowering agents used for treating hyperlipidemic patients. They have a high safety and efficacy profile, yet they have several side effects. All side effects are mainly muscle-related complications like myalgia, rhabdomyolysis, and myopathy. Other side effects include hepatotoxicity, proteinuria, rashes, headaches, and gastrointestinal discomfort [43]. The side effects manifest more severely in patients above the age of 80 and in immunocompromised patients. These side effects cause patients to abruptly discontinue the drugs without consulting their physician [44]. This abrupt discontinuation of medication is called statin intolerance. Statin intolerance is identified by gauging the levels of creatine kinase and hepatic transaminases. Statin-induced elevation in creatine kinase levels is a good indicator of myopathy, which may progress to rhabdomyolysis at higher doses of statins. Patients with a creatine kinase level more than 10 times the upper limit of normal (ULN) are a strong indicator of myopathy [45].

At baseline doses of statins, there is a very rare elevation of hepatic enzymes, but when higher doses are given, the increase in enzymes varies with different statins. The use of statins is contraindicated in patients with active liver diseases [46]. On routine examination, if alanine aminotransferase and aspartate aminotransferase are found to be more than 3 times the ULN, then statins must be immediately stopped until liver enzymes return to normal levels. Management of statin intolerance includes strategies like switching therapies, alternating dosages, non-statin drugs, lipid-lowering nutraceuticals, and other therapies [47]. Table 2 provides information on the studies analyzed in this systematic review.

**Table 2:** Findings from different sources featured in this systematic review HDL: High-density lipoprotein, LDL: Low-density lipoprotein, VLDL: Very low-density lipoprotein, IDL: Intermediate density lipoprotein

Author	Year	Findings
Mcnamara [1]	1992	Effects of dietary fatty acid on the metabolism of plasma proteins like chylomicrons, LDL, VLDL, HDL, and understanding of dietary fatty acids and their effect on cardiovascular diseases.
Després et al. [2]	1990	Body fat distribution and their effects on the development of cardiovascular diseases.
Schaefer [3]	1997	Role of cholesterol in the management of LDLs and association between dietary fibers and fatty acids.
Chait et al. [4]	2016	Types of lipoproteins, their classification and differentiation.
Gunawan et al. [5]	2021	Effects of dietary fibers on apolipoproteins and risks of cardiovascular diseases
Tomkin et al. [6]	2011	Formation of chylomicrons from cholesterol in the body and their metabolism.
Banerjee et al. [7]	2023	Formation of atheromatous plaque due to poor management of cholesterol levels and disruption of chylomicrons.
Gordon et al. [8]	1989	Protective effects of HDLs and their role in lowering risks of cardiovascular diseases.
Wilson [9]	1990	Low levels of HDLs lead to higher levels of mortality in older age groups.
Hausenloy al. [10]	2008	Raising HDL levels significantly lowers the risk of cardiovascular disease.
Kosmas et al. [11]	2018	Functionality of HDLs and their inverse relation to cardiovascular diseases.
Campos et al. [12]	1992	Low-density lipoproteins, their structure, and prevalence in cardiovascular diseases.
Rizzo et al. [13]	2006	Role of size and quantity of LDLs in the prediction of cardiovascular diseases.
Toth et al. [14]	2014	Higher LDL levels are associated with risks of cardiovascular diseases and require aggressive treatment.
Balz [15]	1995	Oxidation of LDLs and its benefits.
Tatami et al. [16]	1981	Intermediate-density lipoproteins together with LDLs pose a high risk to the cardiovascular system.
Shoji et al. [17]	1998	Formation of IDLs and increased risk of cardiovascular diseases in patients with other morbid conditions like renal failure.
Krauss et al. [18]	1987	Correlation between high levels of IDLs and cardiovascular diseases.
Pechlaner et al. [19]	2017	Very low-density lipoproteins, and their effects on the cardiovascular system.
Eisenberg et al. [20]	1973	Metabolism of VLDLs.
Beer et al. [21]	1982	How high levels of VLDLs contribute to the development of cardiovascular diseases.
Hirayama et al. [22]	2012	Effects of small dense LDLs in the development of cardiovascular diseases and their management.
Cromwell et al. [23]	2007	Formation of plaques in arteries due to thrombogenic action on deposited lipoproteins.
Després [24]	2007	How excess visceral abdominal tissue and LDLs increase the risk of cardiovascular diseases in diabetic patients.
Sacks et al. [25]	2003	Formation of small-sized dense lipoproteins and how it leads to the formation of coronary artery diseases.
Ference et al. [26]	2017	High LDLs cause atherosclerotic cardiovascular diseases.
Navab et al. [27]	2011	Different mechanisms by which HDLs reduce the risk of atherosclerosis and other cardiovascular diseases.
Rader et al. [28]	2014	Anti-oxidant property of HDLs and how it lowers the risk of cardiovascular diseases.
Nitschke et al. [29]	2005	Higher levels of HDLs promote healthier arteries and reduce plaque formation.
Cuchel et al. [30]	2001	Correlation between HDLs and thrombosis.
Huang et al. [31]	2022	Pathological role of VLDLs and how they benefit the cardiovascular system.
Lawler et al. [32]	2017	Lowering the levels of VLDLs to help reduce the risks of coronary artery diseases.
Vaziri [33]	2014	Conversion of VLDLs to LDLs and related risks in patients with chronic kidney diseases.
Nordestgaard et al. [34]	2014	Risks of high levels of VLDLs.
Simons et al. [35]	1987	Cholesterol remnants specifically chylomicrons lead to increased risks of cardiovascular diseases.
Ginsberg et al. [36]	2021	Metabolism of chylomicrons releases fatty acids and glycerol. Excess of these increases the risk of cardiovascular diseases.
Weintraub et al. [37]	1996	Patients with coronary artery diseases had increased levels of chylomicrons in blood plasma even though plasma lipid levels were normal.
Xiao et al. [38]	2012	Regulation of chylomicrons and risks and complications due to high levels.
Mann et al. [39]	2014	Benefits of exercise in regulating levels of cholesterol and preventing atherosclerosis.
Williams 40]	2008	Weight management and its importance in regulating cholesterol levels.
Kokkinos et al. [41]	1999	Physical activity and its benefits in lowering co-morbidities which can lead to cardiovascular diseases.
Pahan [42]	2006	Effects and benefits of medications in control and prevention of cholesterol levels.
Bełtowski et al. [43]	2009	Various side effects and adverse drug reactions occurring in patients who are on statins.
Gotto Jr [44]	2006	Research on statins as a lipid-lowering agent and the role of physicians and patients in deciding a proper treatment using statins.
Armitage [45]	2007	Different side effects of statins as a lipid-lowering agent. How elevated levels of creatine kinase help identify statin intolerance.
Argo et al. [46]	2008	Use of statins a lipid-lowering agent and its side effects. Statins' side effects cause an increase in liver enzymes.
Hansen et al. [47]	2005	Different ways of managing statin-induced side effects and statin intolerance in patients.

## Conclusions

Lipoproteins play a crucial role in the transport and management of cholesterol and, in turn, significantly impact the cardiovascular system. The HDLs benefit as they help regulate excess cholesterol in the bloodstream, reducing the risk of atherosclerosis and other heart diseases. On the other hand, LDLs become harmful in excess, causing the accumulation and deposition of cholesterol in arterial walls that lead to plaque formation, thus increasing the risk of cardiovascular events. Maintaining a balance between HDLs and LDLs is essential for maintaining a healthy cardiovascular system. Lifestyle factors, which include a healthy diet, regular exercise, and avoiding smoking, positively affect lipoprotein levels. Medications are prescribed to control cholesterol levels if lifestyle changes are insufficient. Research related to lipoproteins and their effects on the cardiovascular system continues to progress and provides valuable information about the mechanisms of heart disease and possible treatment measures. By understanding the complex relationship between lipoproteins and the cardiovascular system and the harm and benefit of each lipoprotein, healthcare professionals can better assess and manage cardiovascular risk, ultimately promoting a healthy heart and reducing the burden of cardiovascular disease.
